# Genetic variation in RYR1 is associated with heart failure progression and mortality in a diverse patient population

**DOI:** 10.3389/fcvm.2025.1529114

**Published:** 2025-02-21

**Authors:** Leonardo A. Guerra, Christelle Lteif, Yimei Huang, Rylie M. Flohr, Alejandra C. Nogueira, Brian E. Gawronski, Julio D. Duarte

**Affiliations:** ^1^ Center for Pharmacogenomics and Precision Medicine, Department of Pharmacotherapy and Translational Research, University of Florida College of Pharmacy, Gainesville, FL, United States; ^2^ Center For Integrative Cardiovascular and Metabolic Disease, University of Florida, Gainesville, FL, United States; ^3^ Department of Pharmacy, UF Health Shands Hospital, Gainesville, FL, United States

**Keywords:** RYR1, heart failure, survival, arrhythmias, single nucleotide polymorphisms, gene expression

## Abstract

**Introduction:**

Heart failure (HF) is a highly prevalent disease affecting roughly 7 million Americans. A transcriptome-wide analysis revealed *RYR1* upregulation in HF patients with severe pulmonary hypertension. Therefore, we aimed to further characterize the role of *RYR1* in HF progression and mortality.

**Methods:**

In a mouse model of HF, expression of *Ryr1* was compared in cardiac pulmonary, and vascular tissue between HF and control mice. Candidate single nucleotide polymorphisms (SNPs) in the *RYR1* gene region were identified, including variants affecting *RYR1* expression in relevant tissue types. A Cox proportional hazard model was used to analyze genetic associations of candidate SNPs with all-cause mortality in HF patients. An exploratory analysis assessed significantly associated SNPs with risk of HF and arrhythmia development.

**Results:**

In the preclinical HF model, left ventricular expression of *Ryr1* was increased compared to control (fold change = 2.08; *P* = 0.01). In 327 HF patients, decreased mortality risk was associated with two *RYR1* SNPs: rs12974674 (HR: 0.59; 95% CI: 0.40–0.87; *P* = 0.007) and rs2915950 (HR: 0.62, 95% CI: 0.43–0.88; *P* = 0.008). Based on eQTL data, these SNPs were associated with decreased *RYR1* expression in vascular tissue. Two missense variants, in linkage disequilibrium with rs2915950 (rs2915952 and rs2071089) were significantly associated with decreased mortality risk (*P* = 0.03) and decreased risk of atrial fibrillation/flutter (OR: 0.66, 95% CI: 0.44–0.96; *P* = 0.03 and OR: 0.67, 95% CI: 0.45–0.98; *P* = 0.04, respectively). Survival associations with these SNPs were replicated in HF patients self-identifying as Black in the UK Biobank, and the arrhythmia associations were replicated in the overall UK Biobank population.

**Conclusion:**

Increased *RYR1* expression may contribute to HF progression, potentially through the mechanisms associated with calcium handling and arrhythmia development. Our findings suggest that *RYR1* should be further studied as a potential therapeutic target for reducing HF-related mortality.

## Introduction

Heart failure (HF) is a complex and heterogenous condition affecting nearly 7 million American adults, and the disease remains a leading cause of hospitalization and mortality in the United States (1). Over the past 30 years, many treatment advancements have been developed that improve survival; however, the 5-year mortality rate after initial HF hospitalization remains approximately 50% (1). HF is classified into two primary phenotypes: HF with preserved ejection fraction (HFpEF) and HF with reduced ejection fraction (HFrEF). These subtypes differ in their underlying pathophysiology, but disruptions in calcium ion (Ca^2^^+^) signaling occur in both (2–6).

Calcium signaling plays a critical role in cardiac excitation-contraction coupling, and aberrations in Ca^2^^+^ handling contribute to HF progression (7). In particular, disruptions in Ca^2+^ levels within the sarcoplasmic reticulum may result in reduced contraction and relaxation of cardiomyocytes (8). These abnormalities can contribute to systolic dysfunction and ventricular tachyarrhythmias, further compromising cardiac function and eventually leading to myocardial injury (9, 10). This aberrant Ca^2+^ efflux in cardiomyocytes is partially regulated by the ryanodine receptors (RYRs) (11, 12). The three RYR isoforms found in mammals are ryanodine receptor 1 (RYR1), ryanodine receptor 2 (RYR2), and ryanodine receptor 3 (RYR3) (13). RYRs share similar structure and are crucial for calcium release from internal stores in various cell types, including various muscle cell types. Variants in the genes encoding RYR1 and RYR2 can lead to pathologies affecting muscle function (14, 15). Among the three isoforms, RYR2 has been well-studied in the context of HF, as its hyperphosphorylation leads to leaky calcium channel and, seemingly, in severely abnormal calcium exchange in advanced HFpEF and in HF with reduced ejection fraction (HFrEF) (16, 17). RYR1, while primarily associated with skeletal muscle function, has also been implicated in cardiomyocyte Ca^2+^ handling. However, its contribution to aberrant Ca^2+^ movement in the context of HF, including potential differences in HFpEF vs. HFrEF, and cardiac diseases is not as well-established, resulting in limited available data regarding its role in disease progression.

We previously conducted a transcriptome-wide association analysis in HF patients, comparing patients who developed pulmonary hypertension (PH) to those without PH (18). We observed that *RYR1* was significantly upregulated in HF patients with the more severe pre- and post-capillary PH compared to HF patients without PH (18). The precise contribution of RYR1 to HF progression remains unclear. Previous clinical data indicates RYR1 is associated with HF via skeletal muscle sequelae, such as exercise intolerance (19). Further, significantly higher RYR1 protein expression was observed in the left ventricles of patients with end-stage HF compared to nonfailing hearts from organ donors (20). Moreover, both preclinical HF models and clinical data have shown that heightened adrenergic activity leads to the hyperphosphorylation of RYR1, thereby exhibiting altered Ca^2+^ regulation (19, 21–23).

As few studies have assessed the relationship between *RYR1* expression and HF, the aim of this study was to further characterize the role of *RYR1* in HF disease progression using a translational science approach that combines pre-clinical investigations, *in silico* data analysis, and clinical outcomes analysis.

## Materials and methods

### Preclinical HF model

A previously published HF mouse model which replicates key clinical characteristics of HF with PH development was utilized (24). This HF-PH mouse model was previously confirmed to exhibit the same phenotype in our laboratory (18). After weaning, male and female AKR/J mice (Jackson Laboratories, Bar Harbor ME) were randomly assigned to either standard diet (control; 10% kcal fat) or a high-fat diet (HFD; 60% kcal fat; Research Diets, New Brunswick, NJ) for 16–20 weeks. Over this period, the HFD mice develop obesity, metabolic dysregulation, and HFpEF-PH, assessed by left and right heart catheterization, as previously described (24). For this procedure, mice were given tribromoethanol intraperitoneally at approximately 500 mg/kg once (recommended dose for larger mice), and anesthesia plane was confirmed by mouse toe pinch stimulus. After 16–20 weeks on their assigned diet, mice were euthanized by tribromoethanol overdose (approximately 1 g/kg) and subsequent cervical dislocation. Cardiac, pulmonary, and vascular tissues were excised and immediately flash frozen in liquid nitrogen for subsequent analysis. Tribromoethanol was chosen for its minimal cardiovascular effects, which are critical in this model to avoid confounding our findings. Other anesthetics, such as isoflurane, have been associated with significant cardiovascular effects, including alterations in heart rate and blood pressure, which could interfere with the assessment of cardiac function. Tribromoethanol has a relatively short duration of action and, when used for terminal procedures, does not introduce long-term or chronic side effects like abdominal adhesions or ileus, which are typically observed when used repeatedly in survival surgeries. In this study, the drug was administered for terminal procedures in non-pregnant animals and because the animals were euthanized before regaining consciousness, the above-mentioned chronic effects were not observed. Multiple steps were taken to control for potential confounders. First, mice were randomly assigned to diet groups to prevent selection bias. Additionally, both male and female mice were included to account for sex differences. The use of a high-fat diet allowed for the development of obesity and metabolic syndrome, common comorbidities in HFpEF patients, making it an appropriate mouse model that replicates key clinical features of HFpEF (24). The potential influence of medication use was also controlled for by not administering any drugs that could interfere with the study's outcomes during the experimental period. Finally, the animals were euthanized at a pre-defined endpoint (16–20 weeks), ensuring a consistent timeframe for evaluating the effects of diet on the development and progression of HFpEF and PH. All animal procedures were approved by the Institutional Animal Care and Use Committee at the University of Florida and were performed in accordance with the National Institutes of Health Guide for the Care and Use of Laboratory Animals (eighth edition, 2011).

To investigate the potential involvement of *Ryr1* in HF and PH development, cardiac tissue samples were collected from the left ventricle (LV) and right ventricle (RV) of both HF and control mice. Lung tissue was excised to explore the potential involvement of *Ryr1* in the progression of left HF leading to PH, indicating a more severe HF phenotype. Additionally, because of the role of *Ryr1* in calcium-dependent smooth muscle contraction, representative vascular tissue from the descending abdominal aorta was also collected. To determine whether observed associations were specific to *Ryr1*, expression of *Ryr2* (primarily expressed in the heart) was also assessed in cardiac and vascular tissue samples (13).

### Cardiac, pulmonary, and vascular gene expression analysis

Tissues were homogenized, and RNA was isolated. The RNeasy Mini Fibrous Tissue kit (Qiagen Inc., Valencia, CA) was used for cardiac and vascular tissue, while the RNeasy Mini kit (Qiagen Inc., Valencia, CA) was used for pulmonary tissue. Reverse transcription to complementary DNA (cDNA) was done using the High-Capacity cDNA Reverse Transcription Kit (ThermoFisher, Waltham, MA). *Ryr1* and *Ryr2* gene expression was measured via quantitative real-time PCR using TaqMan Gene Expression Assays (assay IDs: Mm01175211_m1 and Mm02619580_g1, respectively; ThermoFisher, Waltham, MA). The comparative 2^−*Δ*Ct^ method was used for calculating relative gene expression of *Ryr1* and *Ryr2* with normalization to the housekeeping gene *Actb* (assay ID: Mm02619580_g1; ThermoFisher, Waltham, MA) and reported as fold-changes relative to the control group (25).

### Study populations

Genetic analyses were first conducted in a discovery cohort of a racially diverse HF population recruited from the cardiology clinics of the University of Illinois Health System (UIC-HF) between November 2001 and September 2015, with follow-up concluding in November 2015, as previously described (26, 27). This comprehensive dataset was compiled by manually extracting information encompassing demographic, medical history, and pharmacotherapy from the electronic health record at the time of enrollment. Blood samples for DNA isolation were also collected. In addition, mortality data were obtained from medical records and the Social Security Death Index. The study was performed according to protocols approved by the University of Illinois at Chicago Institutional Review Board, and all included patients provided written informed consent prior to their participation. The study complied with the Declaration of Helsinki.

The replication cohort consisted of participants recruited in the UK Biobank (UKB) (28). Information on age, sex, self-identified race, and medical history was available. To prevent shifts in survival rates caused by the temporal trends of the COVID-19 pandemic, the date of censoring was set on 01/29/2020, the day of the first reported case of COVID-19 in the UK. Participants were included in the HF subgroup if they had at least 1 documented International Classification of Diseases-10 (ICD10) diagnosis of HF (I50), had the first date of HF diagnosis reported before the censoring date (01/29/2020), were diagnosed with HF after the age of 18, and had not died on the date HF was first reported. This study was conducted using the UKB Resource under application number 97332 and was approved by the University of Florida Institutional Review Board. The de-identified data was provided by UKB. Written informed consent for participation was not required from the participants or the participants' legal guardians in accordance with national legislation and institutional requirements, as no interventions were involved, and no identifiable private data were obtained. This study also complied with the Declaration of Helsinki.

### Outcome measures

The primary outcome of interest was time to all-cause mortality in HF patients. In the UIC-HF discovery cohort, patients who did not die during the follow-up period were censored. Age at censor was defined as the patient's age at their last follow-up or the most recent check of the Social Security Death Index, whichever occurred last. In the UKB HF replication cohort, the follow-up time was defined as the time between the date of the first HF diagnosis and the date of death or censoring, and age at censor or death was calculated accordingly.

For exploratory analyses assessing arrythmias in the discovery cohort, atrial fibrillation/flutter and ventricular tachycardia were analyzed as a composite variable, and separately. In the replication cohort of UKB HF patients, arrhythmias were defined by ICD10 codes (I47, I48, I49), and atrial fibrillation/flutter (I48) was also analyzed separately.

### Patient genotyping, RYR1 single nucleotide polymorphism selection, and functional mapping of associated SNPs in RYR1

For the UIC-HF discovery cohort, whole blood samples were collected and stored at −80 °C at the time of enrollment. DNA isolation was performed and genotyping was carried out utilizing the Affymetrix Axiom PanAfrican Array (ThermoFisher Scientific, Waltham, MA), as previously described (27). This array selection ensured that the genetic coverage of patients within the racially diverse UIC-HF cohort was optimized. Additionally, genotype imputation was performed using the National Heart, Lung, and Blood Institute Trans-Omics for Precision Medicine (TOPMed) Imputation Server (29, 30). The TOPMed panel consists of individuals residing in the United States with diverse ancestries, making it suitable for the UIC-HF cohort (31). Post-imputation variants with an imputation quality r^2^ < 0.5 were excluded.

To assess the potential impact of genetic variation in *RYR1* on clinical outcomes in HF, a rigorous multi-step process was employed to identify putatively functional candidate single nucleotide polymorphisms (SNPs) using publicly available *in silico* data. Initially, a batch query of the entire *RYR1* gene region was performed using SNPnexus (32, 33). From this query, all known SNPs within the region were extracted, and filtering was conducted to retain only non-synonymous variants, as defined by dbSNP, due to their potential impact on protein function (34). Additionally, SNPs functioning as expression quantitative trait loci (eQTLs) for *RYR1* were identified using data from the GTEx Portal (release v8; human genome build 38) (35). To ensure the selection of strong candidate SNPs, only those that served as eQTLs in more than one relevant tissue, including heart, lung, and vascular tissue, were retained.

After the initial list of candidate SNPs was compiled, genotypes for these SNPs were extracted from the study population. Linkage disequilibrium (LD) pruning was subsequently applied using PLINK, with SNPs exhibiting a pairwise correlation coefficient (r^2^) greater than 0.8 being excluded to reduce redundancy (36). Finally, SNPs with minor allele frequencies (MAF) below 0.05 were filtered out to ensure sufficient representation in the population for robust statistical analysis. By applying this systematic approach, a refined list of putatively functional candidate SNPs in *RYR1* was identified for downstream analyses.

The imputed genotype data of the replication cohort, completed and released by the UKB, was further processed for quality control purposes (28). SNPs were filtered out if their imputation quality r^2^ was below 0.5, if their call rate missingness was above 5%, and if their minor allele frequency was below 0.1%. Samples were excluded if they were outliers of extreme heterozygosity or genotype missingness, had per sample genotype call rate missingness above 5%, had a mismatch between self-identified and genotype-inferred sex, or had sex chromosome aneuploidy.

To better pinpoint the most likely functional variants in *RYR1* associated with observed clinical outcomes in HF patients, any missense variants from our original *RYR1* SNP list described above that were LD pruned and in sufficiently high LD (r^2^ > 0.8) with any significant signals from our primary survival analysis were identified and analyzed.

### Statistical analysis

The preclinical phase of the study was prospectively designed to ensure 90% power in detecting a minimum gene expression fold-change of 1.5 at *α* ≤ 0.05. To achieve this, at least 7 mice per group were needed. Tissue gene expression levels were compared between HF and control mice using a two-sample Wilcoxon Rank-Sum test.

In the clinical analyses, candidate SNP deviation from Hardy-Weinberg Equilibrium was tested via a chi-squared test. Survival analyses in the HF patient cohorts were conducted using a multivariable Cox proportional hazard regression model with hazard ratios (HRs) and 95% confidence intervals (CIs) reported. The time from study enrollment to the occurrence of an event (i.e., death) or censoring, was used for time-to-event calculations. In the UIC-HF discovery cohort, the regression model was adjusted for age at death or censoring, sex, self-reported race, New York Heart Association (NYHA) Functional Class, dosage levels of beta-blockers (BBs), dosage levels of angiotensin-converting enzyme inhibitors (ACEIs) or angiotensin receptor blockers (ARBs), aldosterone receptor antagonist (ARA) use, statin use, nitrate use, hydralazine use, potassium supplement use, and history of implantable cardioverter-defibrillator. During the observation period, angiotensin receptor/neprilysin inhibitors and sodium-glucose cotransporter 2 inhibitors were not yet guideline recommended; therefore, data on these medications was not collected. Additionally, relevant clinical factors such as systolic blood pressure, history of ischemic cardiomyopathy, history of atrial fibrillation/flutter, creatinine clearance, smoking history, history of type 2 diabetes mellitus, obesity (body mass index ≥ 30), and serum sodium levels were included in the model (26, 27). The SNP associations were evaluated assuming an additive model (A/A vs. A/a vs. a/a), with the primary outcome being all-cause mortality. To account for multiple SNPs being tested in UIC-HF, *P*-values were adjusted using the false discovery rate (FDR) method, with a FDR threshold of ≤ 0.05 considered statistically significant.

A sensitivity analysis was conducted to verify the consistency of overall results by race. Since Black patients comprised the majority of the population, survival analyses in Black and non-Black subgroups were performed separately, using the same statistical model used in the overall HF population described above. Additionally, a second sensitivity analysis was performed in HFpEF and HFrEF subgroups using the same methods to assess if the observed effects were consistent between different HF types.

As a secondary analysis, SNPs functionally mapped to signals from the primary analysis results underwent survival analyses using the same regression model described in the primary clinical analysis above. A *P*-value ≤ 0.05 was considered statistically significant.

Candidate and functionally mapped SNPs that were significantly associated with the primary outcome in the UIC-HF discovery cohort were analyzed in the UKB HF replication cohort. In the replication cohort, the Cox proportional hazard regression model was adjusted for age at death or censor, sex, self-reported race, smoking status, use of BBs, use of ACEIs/ARBs, and use of ARAs. A sensitivity analysis was also conducted in different racial groups (White, Black, and Asian) to assess consistency and compare with the results from our mostly Black population in UIC-HF. To validate the proportional hazard assumption for the survival analyses, a set of scaled Schoenfeld residuals with a time transformation was utilized (37). Survival curves were estimated to compare the survival distributions of patients.

An exploratory analysis was conducted to evaluate the potential link of the *RYR1* genetic variants associated with the primary outcome in relation to other clinical outcomes in HF patients. Given the role of RYR1 in calcium handling, significantly associated SNPs from the primary analysis were analyzed for their association with tachyarrhythmias, which may influence the risk of developing HF (38), as well as with mortality risk in patients already diagnosed with HF. To better understand if associations between *RYR1* variants and arrhythmia risk were consistent regardless of HF status, the same analyses were also conducted in the overall UKB population. A logistic regression model was used to examine the association between *RYR1* variants and the incidence of arrhythmias adjusting for age at recruitment, sex, race, and smoking status. Odds ratios (ORs) were reported with 95% CIs.

Moreover, the association of *RYR1* SNPs with HF incidence was analyzed in the overall population to assess whether they play a role in arrhythmias alone, or are associated with both arrhythmias and HF development. A logistic regression model was used and adjusted for age at recruitment, sex, race, smoking status, hypertension, type 2 diabetes, coronary artery disease, myocardial infarction, and atrial fibrillation/flutter. A *P*-value ≤ 0.05 was considered statistically significant for replication and exploratory analyses. All statistical analyses were performed using R version 4.0.3.

## Results

### Ryr1 expression in cardiac, pulmonary, and vascular tissue

**Figure 1:**
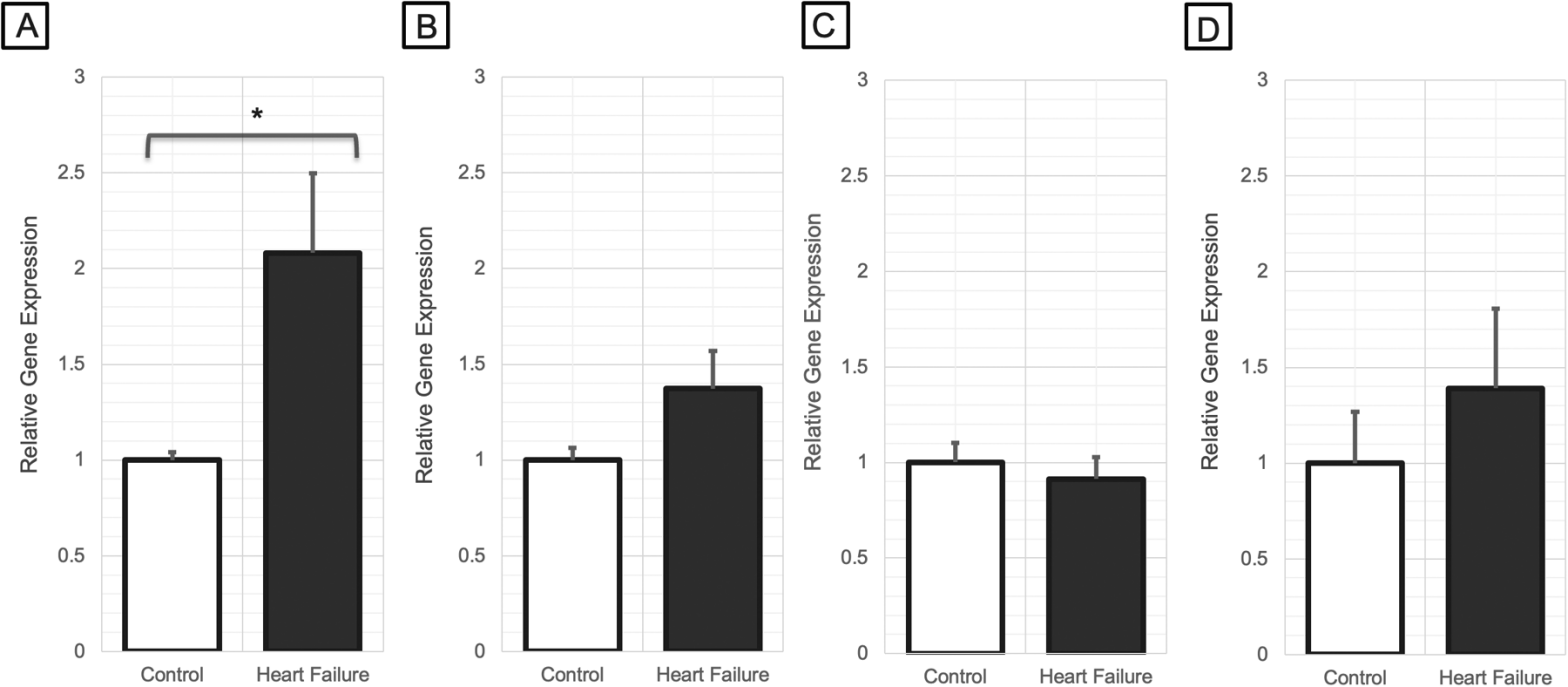
Relative gene expression changes of *Ryr1* in **(A)** left ventricular myocardial tissue (*N* = 7–9/group), **(B)** right ventricular myocardial tissue (*N* = 7/group), **(C)** pulmonary tissue (*N* = 8/group), and **(D)** arterial vascular tissue (*N* = 6-7/group) of heart failure mice vs. control mice (*P < 0.05). *P*-values were computed using a Wilcoxon rank sum test to compare the 2^−*Δ*Ct^ values between the groups.

Compared to control mice, *Ryr1* expression was significantly higher in the LV of HF mice (fold change = 2.08; *P* = 0.01; Figure 1A). However, no significant differences in *Ryr1* expression were observed in the RV (*P* = 0.21; Figure 1B), pulmonary tissue (*P* = 0.44; Figure 1C), or vascular tissue (*P* = 0.18; Figure 1D). There were no significant differences in *Ryr2* expression between HF and control mice in any of the tissues analyzed (Supplementary Figure S1).

### UIC-HF patient demographics

A total of 327 patients were available for analysis in the discovery cohort. Among these patients, 204 (62.4%) were diagnosed with HFrEF, while 123 (37.6%) were diagnosed with HFpEF (Table 1). Overall, 85 of 327 (25.9%) patients died during a median follow-up of 2.8 years (interquartile range: 3.4 years). The racial composition of the study population was diverse, comprising approximately of 73% Black, 16% Latinos, 10% Non-Hispanic White, and 1% Asian (Table 1). As for arrhythmias, 26% of patients had atrial fibrillation/flutter and 16.5% had ventricular tachycardia. Detailed medication use and other characteristics are shown in Table 1.

**Table 1:** UIC-HF cohort baseline characteristics.

Variable	*N* = 327
Age at Event/Censor (years)	62.9 ± 13.2
Female (%)	49.8
Self-reported Race/Ethnicity	
Black (%)	73.4
Non-Latino White (%)	10.1
Asian (%)	0.9
Hispanic/Latino (%)	15.6
NYHA Functional Class (%)	
I	25.4
II	30.0
III	41.9
IV	1.5
HFpEF Diagnosis (%)	37.6
Ischemic Cardiomyopathy (%)	28.7
Atrial Fibrillation/Flutter (%)	26.0
Ventricular Tachycardia (%)	16.5
Type 2 Diabetes (%)	50.2
Obesity (%)	62.4
Creatinine Clearance (ml/min)	92.1 ± 53.1
History of Hypertension (%)	89.3
Smoking Status	
Current Smoker (%)	42.2
Past Smoker (%)	16.8
Never Smoker (%)	40.4
ACEI/ARB Use (%)	89.6
BB Use (%)	96.3
Loop Diuretic Use (%)	78.9
Statin Use (%)	64.2
ARA Use (%)	19.3
Nitrate and Hydralazine Use (%)	34.6
Potassium Supplement Use (%)	21.7

All variables are reported as mean ± SD or %. The ± symbol represents the standard deviation, indicating how much the data points deviate from the mean.

ARA, aldosterone receptor antagonist; ACEI, angiotensin-converting enzyme inhibitor; ARB, angiotensin receptor blocker; BB, beta-blockers; HFpEF, heart failure with preserved ejection fraction; NYHA, New York Heart Association.

### UKB patient demographics

A total of 13,017 patients with genotype data available were included in the HF population as the replication cohort (Table 2). Overall, 4,954 (38.1%) patients died during a median follow-up of 3.4 years (interquartile range: 6.8 years). The majority of patients were White (95.4%), 2.7% were Asian, and 0.4% were Black. Arrhythmias were present in 50.9% of patients, and atrial fibrillation/flutter in 38.8%. Medication use data is provided in Table 2. The total UKB population (479,665 participants) had similar race distribution, with 2.7% diagnosed with HF and 29.9% with hypertension (Supplementary Table S1).

**Table 2:** UKB HF cohort baseline characteristics.

Variable	*N* = 13,017
Age at Recruitment	62.2 ± 6.1
Age at Event/Censor (years)	72.2 ± 6.4
Female (%)	34.3
Self-reported Race/Ethnicity	
White (%)	95.4
Black (%)	0.4
Asian (%)	2.8
Mix/other (%)	0.9
Atrial Fibrillation/Flutter (%)	38.8
Arrhythmias (%)	50.9
Smoking Status	
Current Smoker (%)	15.2
Past Smoker (%)	46.2
Never Smoker (%)	37.9
ACEI/ARB Use (%)	47.7
BB Use (%)	31.5
ARA Use (%)	3.9

All variables are reported as mean ± SD or %. The ± symbol represents the standard deviation, indicating how much the data points deviate from the mean.

ARA, aldosterone receptor antagonist; ACEI, angiotensin-converting enzyme inhibitor; ARB, angiotensin receptor blocker; BB, beta-blockers.

### RYR1 survival analysis

**Figure 2:**
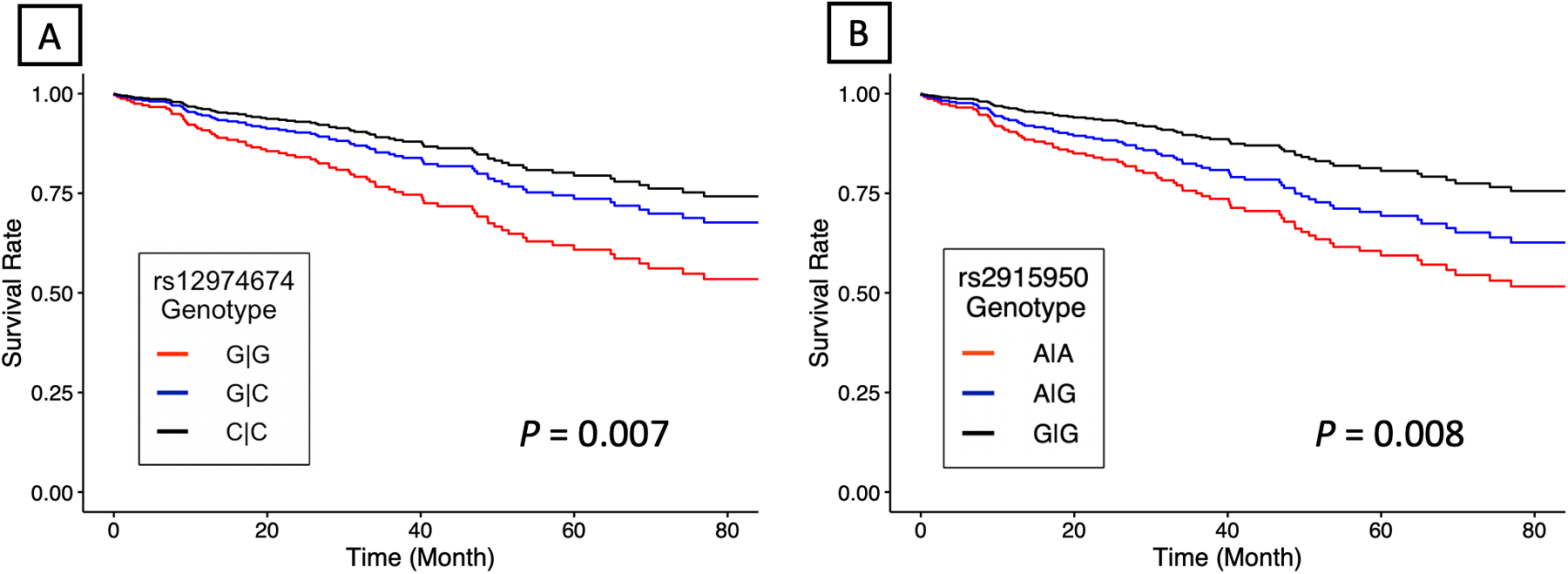
Estimated adjusted hazard curves for *RYR1* single nucleotide polymorphisms. **(A)** rs12974674 (*N* = 156 for G/G, *N* = 130 for G/C, and *N* = 41 for C/C) and **(B)** rs2915950 (*N* = 64 for G/G, *N* = 148 for G/A, and *N* = 115 for A/A) genotype associations with all-cause mortality. Lines denote the homozygous common genotype (red), the heterozygous genotype (blue) and the homozygous variant genotype (black), respectively. *P*-values were computed using a multivariable Cox proportional hazard regression model.

**Figure 3:**
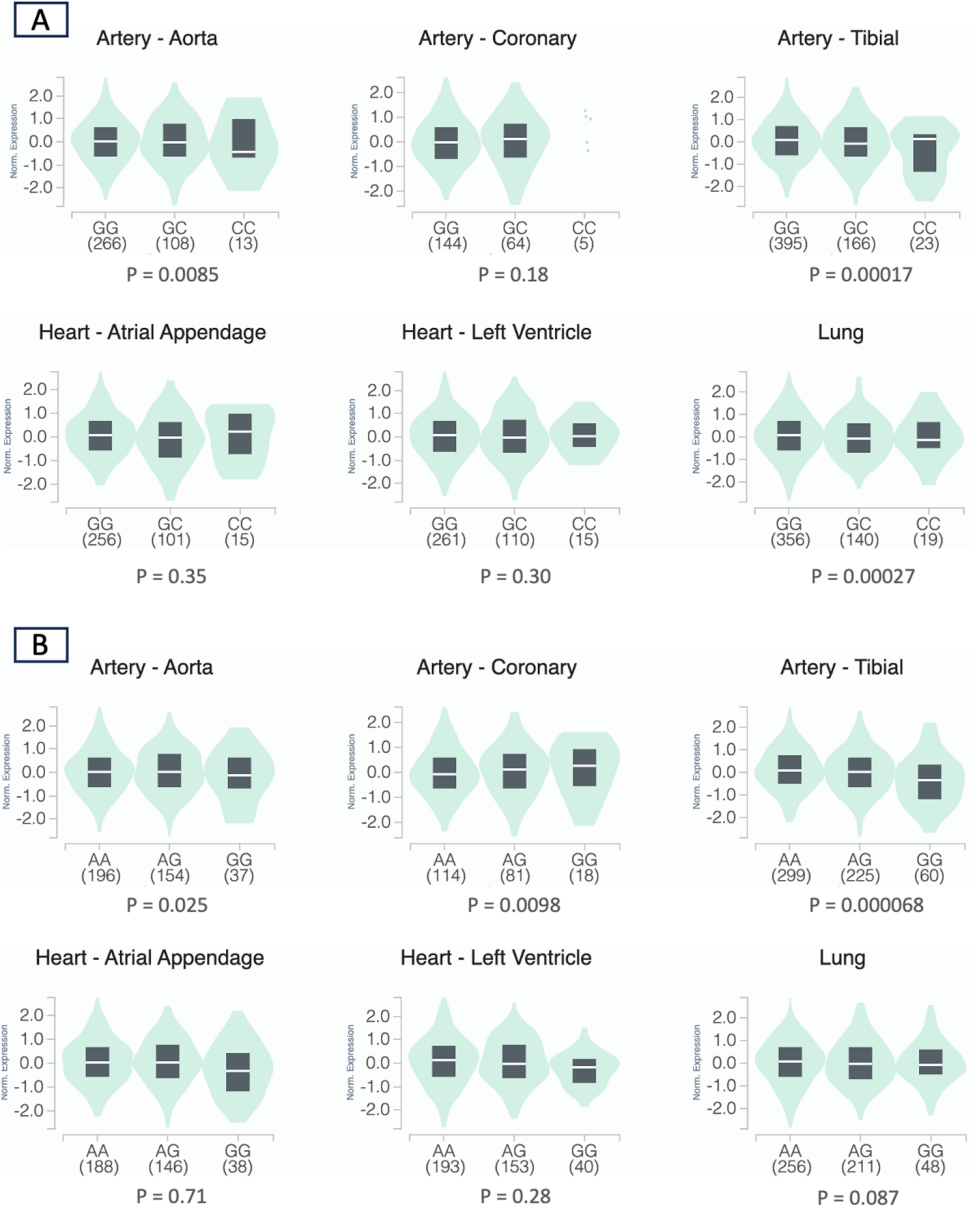
Violin plots depicting the normalized expression levels of *RYR1* by rs12974674 **(A)** and rs2915950 **(B)** genotype using data obtained from the GTEx database (human genome build 38) (20). For rs12974674, the G and C alleles represent the major and minor alleles, respectively, and for rs2915950, the A and G alleles represent the major and minor alleles, respectively. The density distribution of samples within each genotype is visualized in green, while the median expression value of *RYR1* for each genotype is represented by the white line within the black box plot. Sample sizes for each genotype are included in the parentheses under the corresponding genotype.

The initial list of SNPs in/near *RYR1* contained 859 variants. After screening and LD pruning, 10 candidate SNPs were identified for clinical association analysis (Supplementary Table S2). All 10 candidate SNPs were eQTLs for *RYR1*. Of these SNPs, two were significantly associated with mortality after adjusting for multiple testing. Notably, variation at rs12974674 was significantly associated with reduced mortality risk (HR: 0.59, 95% CI: 0.40–0.87; *P* = 0.007), with the homozygous variant (CC) genotype possessing the lowest risk (Figure 2A; Supplementary Tables S2 and 3). Another candidate SNP, rs2915950, showed a similar association with a decreased mortality risk (HR: 0.62, 95% CI: 0.43–0.88; *P* = 0.008), with the greatest survival observed in those with the homozygous variant (GG) genotype (Figure 2B; Supplementary Table S2 and S3). These two SNPs were in only moderate LD (r^2^ = 0.47). Of the remaining eight SNPs that were analyzed, only rs2907616 was similarly associated with a decrease in mortality risk (HR: 0.68, 95% CI:0.48–0.98; *P* = 0.04), but this association did not remain significant after FDR adjustment (Supplementary Table S2). According to GTEx data, the variant alleles of both rs12974674 and rs2915950 are associated with reduced *RYR1* expression in most of the vasculature data available (particularly in the aorta and tibial artery), and rs12974674 is also associated with decreased expression of *RYR1* in pulmonary tissue (Figure 3).

The first sensitivity analysis was conducted by race. In the subgroup of black patients (*N* = 240), rs12974674 was associated with decreased risk of all-cause mortality (HR = 0.56, 95% CI: 0.35–0.89, *P* = 0.01), similar to the overall cohort. In the smaller non-Black subset (*N* = 87), rs12974674 had a similar direction of effect with all-cause mortality, but the association was no longer statistically significant (HR = 0.51, 95% CI: 0.21–1.24, *P* = 0.14). A mortality trend consistent with the overall cohort was also observed for rs2915950 in the Black subset; however, it did not reach statistical significance (HR = 0.67, 95% CI: 0.43–1.05, *P* = 0.08). Further, rs2915950 was significantly associated with increased survival in the non-Black subset (HR = 0.46, 95% CI: 0.21–0.99, *P* = 0.05).

In the second sensitivity analysis in specific HF subtypes, we tested the impact of the significantly associated SNPs in HFrEF and HFpEF patients separately. In the HFrEF subgroup, both rs12974674 (HR: 0.53, 95% CI: 0.29–0.97; *P* = 0.04) and rs2915950 (HR: 0.47, 95% CI: 0.26–0.84; *P* = 0.01) remained significantly associated with decreased mortality risk. In the HFpEF subgroup, rs12974674 continued to demonstrate a significant association with decreased mortality risk (HR: 0.51, 95% CI: 0.27–0.95; *P* = 0.033), while the association for rs2915950 remained directionally similar but did not reach statistical significance (HR: 0.68, 95% CI: 0.39–1.19; *P* = 0.18).

After additional functional mapping of the associated candidate SNPs, we identified two missense variants in *RYR1* in high LD with rs2915950: rs2915952 (r^2^ = 0.87) and rs2071089 (r^2^ = 0.86). Both rs2915952 (HR: 0.66, 95% CI: 0.45–0.96; *P* = 0.03) and rs2071089 (HR: 0.66, 95% CI: 0.45–0.96; *P* = 0.03) were similarly associated with reduced mortality risk. Additionally, GTEx data indicated that the variant alleles of both rs2915952 and rs2071089 were associated with decreased *RYR1* expression in most of the vasculature data available (particularly in the aorta and tibial artery), as observed with rs2915950 (Supplementary Figures 2 and 3). No functional variants were identified in similarly high LD with rs12974674.

In the replication UKB HF cohort, none of the SNPs were significantly associated with mortality in the overall population. However, all four SNPs replicated associations with reduced risk of death in the subgroup of participants identifying as Black (*N* = 56): rs12974674 (HR: 0.41, 95% CI: 0.17–0.99; *P* = 0.05), rs2915950 (HR: 0.50, 95% CI: 0.25–1.00; *P* = 0.05), rs2915952 (HR: 0.46, 95% CI: 0.21–0.99; *P* = 0.05), and rs2071089 (HR: 0.46, 95% CI: 0.21–1.00; *P* = 0.05).

### Exploratory associations with arrhythmias and HF incidence

In our exploratory analysis of arrhythmia risk in the UIC-HF discovery cohort, we found no significant association between the SNPs and the occurrence of tachyarrhythmias overall. However, we found significant associations between decreased risk of atrial fibrillation/flutter and both rs2915952 (OR: 0.66, 95% CI: 0.44–0.96; *P* = 0.03) and rs2071089 (OR: 0.67, 95% CI: 0.45–0.98; *P* = 0.04).

We found no significant associations with arrhythmias or atrial fibrillation/flutter in the HF subgroup of the UKB replication cohort. However, we observed significant associations between decreased risk of atrial fibrillation/flutter and rs2915950 (OR: 0.96, 95% CI: 0.94–0.98; *P* = 4.46 × 10^−4^), rs2915952 (OR: 0.96, 95% CI: 0.94–0.98; *P* = 8.16 × 10^−4^), and rs2071089 (OR: 0.97, 95% CI: 0.94–0.99; *P* = 1.23 × 10^−3^) in the overall UKB population. Finally, we also observed significant associations between reduced risk of HF development and both rs2915952 (OR: 0.97, 95% CI: 0.94–1.00; *P* = 0.05) and rs2071089 (OR: 0.97, 95% CI: 0.94–1.00; *P* = 0.04) in the overall UKB population.

## Discussion

In this translational study, we explored the link between *RYR1*, HF progression, and mortality. In our previous work, we noted a significant upregulation of *RYR1* expression in the blood of HF patients with more severe PH compared to HF patients without PH (18). The development of PH signifies an advancement in HF progression, as indicated by the additional increased mortality risk PH confers in HF patients (39). In this study, we extend these findings by demonstrating a similar increase in *Ryr1* expression within the LV of HF mice. Furthermore, we provide evidence that genetic variants in *RYR1* that are correlated with reduced *RYR1* expression are also associated with improved survival in HF patients and potentially decreased risk of developing atrial fibrillation/flutter. These findings support that *RYR1* may drive (rather than be a response to) the progression of HF because these genetic variants are present from birth.

RYRs have been implicated in abnormal calcium movement, which occurs through receptor phosphorylation, and can affect a variety of tissue types (11, 12, 16, 17). Previous RYR research in HF has primarily indicated involvement of *RYR2* in disease progression (40), but our results suggest that *RYR1* is also involved. While RYR2 is predominantly expressed in cardiac muscle and activated by calcium-induced calcium release, RYR1 is primarily found in skeletal muscle, with lower levels in vascular smooth muscle cells, and is activated by mechanical coupling with the dihydropyridine receptor. RYR2 also shows higher sensitivity to calcium activation compared to RYR1 (13, 41, 42). These distinctions extend to their associated disorders, with *RYR2* mutations linked to cardiac arrhythmias such as catecholaminergic polymorphic ventricular tachycardia, while *RYR1* mutations are typically associated with malignant hyperthermia and muscular pathologies (13, 43, 44). While the known biological role of *RYR1* is centered around calcium regulation in skeletal muscle, it also plays a lesser-known role in other tissue types such as smooth muscle, intercalated discs, B-lymphocytes, and cardiac mitochondria (20, 23, 45–47). Studies have shown that localization of RYR1 in cardiomyocyte mitochondria allows for activation to occur promptly upon the release of Ca^2+^ from the sarcoplasmic reticulum (48, 49). Subsequent increases past the maximal local Ca^2+^ levels result in RYR1 inactivation, preventing mitochondrial Ca^2+^ overload (50, 51). Excessive calcium levels within the mitochondria disrupts the membrane potential, and this could impair oxidative phosphorylation and escalate the production of reactive oxygen species which is a prominent factor closely linked to HF (50, 52).

In this study, we found increased *Ryr1* expression, but no changes in *Ryr2*, in the LV of HF mice. These results are consistent with a prior clinical observational study by Münch et al. reporting significantly increased protein expression of RYR1 in the LV of patients with dilated cardiomyopathy and NYHA class IV HF when compared to nonfailing control hearts (20). Additionally, we did not observe any significant association with *Ryr1* expression in RVs of HF mice, which is also consistent with the clinical observations in RVs also reported by Münch et al. (20) While our approach may have captured RNA from both the myocardium and peripheral cardiac vessels, this could provide additional insights into the broader cardiac microenvironment. While the *RYR1* SNPs associated in our clinical analysis seemed to be consistently associated with *RYR1* expression in vascular tissue, we only observed a non-significant increase in vascular tissue. This could be due to greater heterogeneity in the vascular tissue samples (including smooth muscle, endothelial, epithelial, etc.) compared to LV samples or perhaps differences in interspecies *Ryr1* tissue expression patterns. Additional studies would clarify the pre-clinical model generalizability of expression patterns in specific cell types.

This alignment between the observed increased *Ryr1* expression in our animal model and prior reported clinical observations, by our group and others, combined with the absence of observed differential *Ryr2* expression further support that the observed relationship with HF progression is likely specific to RYR1 and does not necessarily extend to RYR2. In a previous study by Kushnir et al*.* including a HF mouse model and patients with HF, the presence of remodeled leaky RYR1 channels was observed in circulating B-lymphocytes, resulting in intracellular Ca^2+^ leak (23). It is reasonable to hypothesize that a similar Ca^2+^ leak may occur in vascular and myocardial tissues, including cardiomyocytes. However, additional research is needed to directly assess the effects of varying levels of RYR1 expression/function, particularly in vascular smooth muscle and cardiomyocytes.

In our clinical data, we identified two SNPs in *RYR1*, rs12974674 and rs2915950, that were associated with improved survival in HF patients. These SNPs are eQTLs for *RYR1*, correlated with reduced gene expression in multiple vascular tissues. Because the potential mechanism by which these two SNPs would affect *RYR1* expression was not obvious, we conducted a functional mapping analysis that identified two putatively functional SNPs in high LD with rs2915950 (rs2915952 and rs2071089) that were also associated with reduced mortality in patients with HF. These putatively functional SNPs are missense variants and are also correlated with reduced *RYR1* expression in multiple vascular tissues according to GTEx data, but appeared to have slightly weaker mortality associations than rs2915950. Interestingly, these mortality associations were only replicated in the small Black subgroup of the UKB HF population which are consistent with our findings given the majority of the UIC-HF discovery cohort consisted of Black patients. The lack of replication in other ancestral populations suggests that these SNPs may not be the causal SNPs and further functional work may be needed. The racial diversity of our discovery cohort enhances the generalizability of our findings to underrepresented populations, particularly those of African descent, who are often excluded or underrepresented in genetic studies. Additionally, the large size of the replication cohort in UKB, consisting predominantly of individuals of European descent, allowed for robust comparisons across ancestral groups. However, the smaller Black subgroup in UKB presented challenges for statistical power in this group and underscores the importance of including diverse populations in future research. The identified SNPs were common across racial groups; therefore, it remains unclear whether the observed associations in Black patients are influenced by environmental factors, disparities in healthcare received, differences in linkage disequilibrium patterns between ancestral populations, or if this pathway plays a more significant role in HF progression in Black patients than in others (53). More research is needed to identify which may be driving the observed differences in SNP associations between Black and White patients. While our primary analysis in the UIC-HF population suggested the *RYR1* SNP associations were present in a racially diverse patient population, the non-significant associations we observed in the non-Black subgroup in the sensitivity analysis likely indicate that either the smaller subgroup had less power to detect effects, or the role of *RYR1* in HF progression may be more pronounced in patients of African descent. The combination of a racially diverse discovery cohort and a large replication cohort provides a unique strength in assessing the generalizability of our findings. Future studies could build on these findings by investigating additional ancestrally diverse populations to further clarify the potential interactions between genetic variation, ancestry, and HF progression.

Our second sensitivity analysis showed similar associations between the *RYR1* candidate SNPs and survival in both HFrEF and HFpEF subgroups, as seen in the overall HF population. In the HFrEF subgroup, rs12974674 and rs2915950 exhibited significant associations with reduced mortality risk. In the HFpEF subgroup, rs12974674 demonstrated a significant association with decreased mortality risk, while rs2915950 trended in the same direction, it did not achieve statistical significance. However, this could be explained by reduced power due to the smaller sample size, especially in the HFpEF subgroup. Unfortunately, it was not possible to accurately assess HF type in UKB patients, so this sensitivity analysis could not be replicated. While our preclinical model utilized HFpEF to investigate these mechanisms, it is important to acknowledge the differences between HFpEF and HFrEF in calcium ion handling and RyR isoform regulation (3, 54). These differences could have implications for understanding how *RYR1* variants affect HF subtypes, despite their seemingly different etiologies. Our findings suggest that *RYR1* variants may exert a similar effect across HF subtypes; however, further research is warranted to explore the interplay between these genetic variants and the distinct pathophysiological mechanisms of HFrEF and HFpEF.

An exploratory analysis in UIC-HF also showed a decreased risk of atrial fibrillation/flutter with rs2915952 and rs2071089. We did not observe a significant association between specific *RYR1* SNPs and ventricular arrhythmias in the UIC-HF cohort. It is important to note that the group of patients with ventricular tachycardia was relatively small, which may have limited our power to detect a significant difference in this subgroup. Since RYR1 has been implicated in abnormal calcium movement, it is conceivable that it could contribute to altered cardiac muscle function, promoting arrhythmogenic events and exacerbating HF progression (21–23, 38). In fact, rs118192162, another *RYR1* mutation associated with malignant hyperthermia, has been shown to increase catecholamine-induced arrythmias through mitochondrial Ca^2+^ overload (55). However, rs2915952 and rs2071089 did not show a significant association with atrial fibrillation/flutter in the UKB HF population, but were associated with decreased risk of atrial fibrillation/flutter in the overall UKB cohort. These *RYR1* SNPs were also associated with reduced risk of HF development in the same overall population, suggesting a potential mechanism by which RYR1 may influence HF development and progression. However, the observed effect sizes for these associations were small and were likely only detectable given the very large sample size available. Additionally, for a cohort like the UKB, the use of diagnostic codes can introduce some noise when classifying cases and controls, which could affect the reliability of the results. These limitations should be considered when interpreting the findings. Further studies are needed to elucidate the functional consequences of these SNPs and any potential roles they may have in arrythmias and cardiac disease progression. Despite these challenges, we did observe significant associations with atrial fibrillation/flutter, further reinforcing the relevance of RYR1 in cardiac disease progression. While not every analysis yielded significant results, it is crucial to emphasize that all significant findings are consistent across different cohorts and experimental models. This alignment strengthens the potential role of RYR1 in heart failure progression.

In a mouse model of HF, Kushnir et al*.* observed reduced mortality with treatment of a drug that reduces RyR1 Ca^2+^ leak (23). Previous research has suggested that gain-of-function variants in both *RYR1* and *RYR2* lead to increased sensitivity for activation of the channels and decreased ability to remain closed (56, 57). These leaky channels can lead to arrhythmias and noncompaction cardiomyopathy with RYR2 and increased risk of malignant hyperthermia with RYR1 (56–58). On the other hand, *RYR2* loss-of-function variants have also been associated with arrhythmias and *RYR1* loss-of-function has been reported in skeletal myopathies (59–61). Although both gain-of-function and loss-of-function *RYR1* variants seem to be deleterious, the impact of reduced *RYR1* expression (resulting in a moderately reduced overall RYR1 function) in heart disease remains uncertain. The identified *RYR1* SNPs might influence HF progression through altered mitochondrial calcium handling, potentially affecting energy metabolism and oxidative stress, distinct from RYR2's role in sarcoplasmic reticulum calcium release. These genetic variations could possibly confer protective effects by stabilizing RYR1 configuration, reducing pathological calcium leak, and/or preserving myocardial function.

Our study benefits from the convergence of three distinct types of data—patient data, *in silico* eQTL data, and tissue-specific gene expression from a preclinical HF model—which all collectively support a similar conclusion. The clinical survival analysis demonstrated reduced mortality in patients with *RYR1* variants which, based on *in silico* data, are associated with reduced expression of *RYR1* in arterial tissue. These findings align with preclinical data showing significantly increased *Ryr1* expression in LV tissue. The presence of genetic variants acquired prior to birth that are associated with decreased *RYR1* expression, coupled with consistent gene expression data in the tissues of interest, strengthens the support for *RYR1* being involved in the process of HF progression rather than being a result of HF disease progression. The identification of *RYR1* genetic variations associated with HF outcomes opens new potential avenues for translational research. If further validated, these SNPs could potentially be incorporated into risk stratification tools, allowing for more precise prediction of HF progression and outcomes. Furthermore, understanding the role of RYR1 in HF could inform the development of novel therapeutic strategies. For instance, therapies targeting RYR1-mediated calcium regulation in cardiac mitochondria might offer a new approach to mitigating oxidative stress and energy metabolism disruptions in HF. Our findings may also have significant implications for precision medicine in HF management. The identified *RYR1* SNPs could serve as genetic markers for subgroups of HF patients who might benefit from tailored treatment approaches, guiding clinicians in selecting the most appropriate therapeutic strategies for individual patients. However, future clinical studies, coupled with a better mechanistic understanding of RYR1, are necessary to evaluate its potential role in personalized HF management.

Our study has some limitations that should be acknowledged. First, the relatively small sample size of our HF patient cohort may have reduced our statistical power to detect more rare *RYR1* genetic associations with smaller effect sizes. Our primary findings were only replicated in a smaller population of Black HF patients. Moreover, our results did not specifically determine the mechanism by which *RYR1* might worsen HF. Additionally, potential biases from population stratification and limited statistical power in subgroup analyses could have influenced the results. However, it is important to note our findings align across various analytical and experimental models employed in our study and also appear consistent with previous studies of RYR1 in HF (18, 20, 23). Further research, including CRISPR-based studies to assess SNP functionality, is needed to validate these findings. Despite the acknowledged limitations, the consistency of our significant associations underscores the potential relevance of RYR1 in the pathophysiology of HF.

## Conclusions

In summary, our findings suggest that RYR1 plays a role in the progression of HF, as supported by both animal studies and patient clinical data. Specific SNPs in Black patients were associated with improved survival, but these associations require further investigation to better understand their biological and clinical significance across different ancestries. Future studies may also aim to determine if *RYR1* expression could serve as a biomarker for HF disease progression or begin exploring the feasibility of targeting RYR1 as a therapeutic strategy for HF.

## Data Availability

The datasets presented in this article are not readily available because the SNP data derived from the UKB is available to researchers upon request through the UK Biobank Access Management System, subject to approval of a data access application and appropriate licensing. The data from the UIC cohort is not readily available as we do not have the necessary permissions or consents from the original participants to make this dataset publicly available. This research has been conducted using the UK Biobank Resource under application number 97332. Further enquiries can be directed to the corresponding author.
