# Supplementary material for: Genetic variation in RYR1 is associated with heart failure progression and mortality in a diverse patient population
Source: Front Cardiovasc Med. 2025 Feb 21;12:1529114. doi: 10.3389/fcvm.2025.1529114 (PMC11885062; doi:10.3389/fcvm.2025.1529114)
Supplement: Supplementary file 1 [file Datasheet1.docx]

Supplementary Material

# Supplementary Figures and Tables

| **Supplementary Table 1:** UKB Overall Cohort Baseline Characteristics. | |
| --- | --- |
| **Variable** | N=479,665 |
| Age at Recruitment | 56.6 ± 8.1 |
| Female (%) | 54.2 |
| Self-reported Race/Ethnicity |  |
| White (%) | 95.5 |
| Black (%) | 0.5 |
| Asian (%) | 2.3 |
| Mix/other (%) | 1.3 |
| HF Diagnosis (%) | 2.7 |
| Myocardial Infarction (%) | 3.3 |
| Atrial Fibrillation/Flutter (%) | 5.2 |
| Arrhythmias (%) | 6.9 |
| Type 2 Diabetes (%) | 7.9 |
| History of Hypertension (%) | 29.9 |
| Smoking Status |  |
| Current Smoker (%) | 10.5 |
| Past Smoker (%) | 34.8 |
| Never Smoker (%) | 54.3 |
| All variables are reported as mean ± SD or %. The ± symbol represents the standard deviation, indicating how much the data points deviate from the mean. | |

| Supplementary Table 2. Summary of polymorphism minor allele frequencies (MAF), all-cause mortality associations, and false discovery rate (FDR) within the UIC-HF discovery cohort. | | | | | | |
| --- | --- | --- | --- | --- | --- | --- |
| SNP | MAF (All) | MAF (European) | MAF (African) | Hazard  Ratio | *P – Value* | FDR |
| rs12974674 | 0.32 (C) | 0.18 (C) | 0.42 (C) | 0.5902 | 0.006866 | 0.04248 |
| rs2915950 | 0.44 (G) | 0.31 (G) | 0.46 (A) | 0.6178 | 0.008496 | 0.04248 |
| rs2907616 | 0.37 (T) | 0.24 (T) | 0.43 (T) | 0.6837 | 0.040762 | 0.1358733 |
| rs2960337 | 0.28 (G) | 0.18 (G) | 0.31 (G) | 0.6559 | 0.08858 | 0.2051667 |
| rs2960351 | 0.25 (T) | 0.01 (T) | 0.37 (T) | 0.7169 | 0.103239 | 0.2051667 |
| rs2960350 | 0.14 (G) | 0.05 (G) | 0.34 (G) | 0.7252 | 0.1231 | 0.2051667 |
| rs115404685 | 0.03 (C) | 0.04 (C) | 0.05 (C) | 0.7066 | 0.39606 | 0.5658 |
| rs115308402 | 0.04 (C) | 0.07 (C) | 0.04 (C) | 0.94 | 0.87096 | 0.99785 |
| rs62120355 | 0.04 (A) | 0.05 (A) | 0.07 (A) | 1.0204 | 0.95388 | 0.99785 |
| rs690825 | 0.04 (T) | 0.002 (T) | 0.13 (T) | 0.9992 | 0.99785 | 0.99785 |

| Supplementary Table 3. Baseline characteristics of the UIC-HF discovery cohort stratified by *RYR1* variant genotypes. | | | | | | | | |
| --- | --- | --- | --- | --- | --- | --- | --- | --- |
| Genotype | rs12974674 (G\|G) | rs12974674 (C\|G) | rs12974674 (C\|C) | P-value | rs2915950 (A\|A) | rs2915950 (G\|A) | rs2915950 (G\|G) | *P*-value |
| Variable | **Value** (N = 156) | **Value** (N = 130) | **Value** (N = 41) |  | **Value** (N = 115) | **Value** (N = 148) | **Value** (N = 64) |  |
| Age at Event/Censor (years) | 63.8 ± 13.4 | 61.5 ± 12.8 | 63.9 ± 13.8 | 0.298 | 64.9 ± 13.5 | 61.9 ± 12.4 | 61.7 ± 14.4 | 0.135 |
| Female (%) | 50.0 | 50.8 | 46.3 | 0.884 | 49.6 | 47.3 | 56.3 | 0.487 |
| NYHA Functional Class (%) |  |  |  | 0.852 |  |  |  | 0.083 |
| I | 25.3 | 28.1 | 19.5 |  | 31.0 | 22.4 | 23.8 |  |
| II | 31.2 | 29.7 | 29.3 |  | 23.0 | 32.0 | 39.7 |  |
| III | 41.6 | 40.6 | 51.2 |  | 42.5 | 44.9 | 36.5 |  |
| IV | 1.9 | 1.6 | 0.0 |  | 3.5 | 0.7 | 0.0 |  |
| HFpEF Diagnosis, (%) | 37.8 | 35.4 | 43.9 | 0.616 | 38.3 | 34.5 | 43.8 | 0.433 |
| Ischemic Cardiomyopathy (%) | 29.7 | 28.7 | 27.5 | 0.959 | 31.6 | 27.4 | 28.1 | 0.751 |
| Atrial Fibrillation/Flutter (%) | 28.8 | 23.1 | 24.4 | 0.525 | 29.6 | 28.4 | 14.1 | 0.051 |
| Type 2 Diabetes (%) | 53.8 | 50.0 | 36.6 | 0.144 | 52.2 | 54.1 | 37.5 | 0.075 |
| Obese (%) | 63.5 | 61.5 | 61.0 | 0.927 | 61.7 | 61.5 | 65.6 | 0.836 |
| Creatinine Clearance (mL/min) | 89.0 ± 50.1 | 96.6 ± 53.5 | 89.5 ± 62.7 | 0.466 | 86.3 ± 45.9 | 91.4 ± 50.9 | 104.2 ± 67.4 | 0.099 |
| History of Hypertension (%) | 89.1 | 87.7 | 95.1 | 0.404 | 85.2 | 90.5 | 93.8 | 0.168 |
| Smoking Status |  |  |  | 0.077 |  |  |  | 0.318 |
| Current Smoker (%) | 37.7 | 43.8 | 56.1 |  | 41.6 | 39.9 | 50.0 |  |
| Past Smoker (%) | 22.1 | 12.3 | 12.2 |  | 13.3 | 20.9 | 14.1 |  |
| Never Smoker (%) | 40.3 | 43.8 | 31.7 |  | 45.1 | 39.2 | 35.9 |  |
| Self-reported Race/Ethnicity |  |  |  | 0.324 |  |  |  | 0.002 |
| Black (%) | 67.3 | 77.7 | 82.9 |  | 60.9 | 79.7 | 81.3 |  |
| Non-Latino White (%) | 13.5 | 7.7 | 4.9 |  | 18.3 | 5.4 | 6.3 |  |
| Asian (%) | 1.3 | 0.8 | 0.0 |  | 0.0 | 1.4 | 1.6 |  |
| Hispanic/Latino (%) | 17.9 | 13.8 | 12.2 |  | 20.9 | 13.5 | 10.9 |  |
| ACEI/ARB Use (%) | 91.4 | 89.9 | 95.1 | 0.839 | 92.8 | 89.2 | 93.7 | 0.490 |
| BB Use, (%) | 96.1 | 97.7 | 92.7 | 0.332 | 98.2 | 95.3 | 95.2 | 0.403 |
| Loop Diuretic Use (%) | 80.5 | 77.5 | 82.9 | 0.703 | 81.4 | 77.7 | 81.0 | 0.730 |
| Statin Use (%) | 69.9 | 58.9 | 65.9 | 0.153 | 66.1 | 64.2 | 65.1 | 0.951 |
| ARA Use (%) | 18.8 | 22.5 | 12.2 | 0.338 | 17.7 | 22.3 | 15.9 | 0.472 |
| Nitrate and Hydralazine Use (%) | 33.3 | 37.7 | 17.1 | 0.975 | 40.0 | 33.8 | 26.6 | 0.676 |
| Potassium Supplement Use (%) | 21.1 | 24.4 | 19.5 | 0.724 | 21.8 | 22.4 | 22.2 | 0.993 |


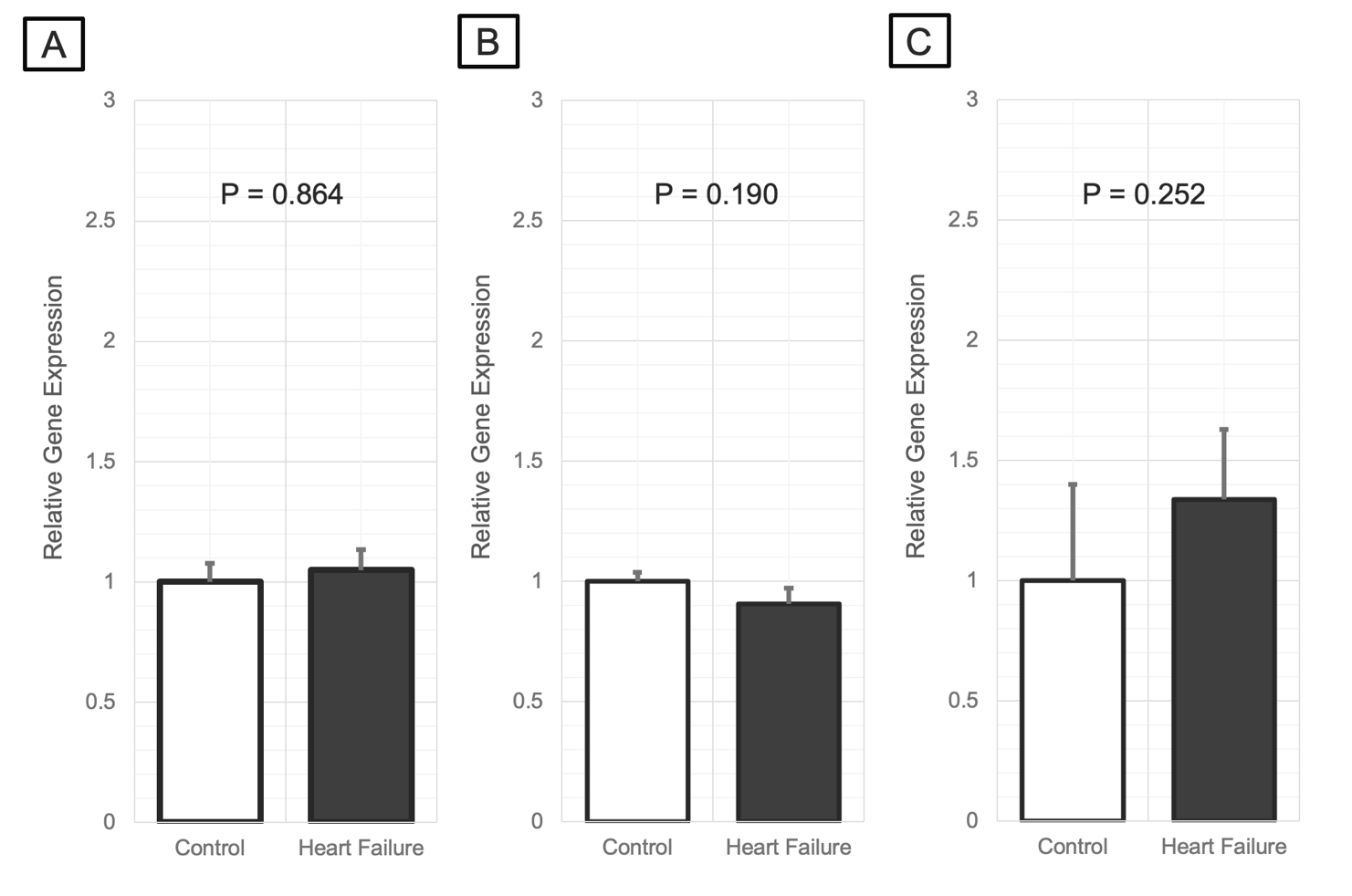


**Supplementary Figure 1**: Relative *Ryr2* expression in (A) left ventricular myocardial tissue (N=6-9/group), (B) right ventricular myocardial tissue (N=9/group), and (C) abdominal aorta (N=7-9/group) from heart failure mice compared with control mice. *P*-values were computed using a Wilcoxon rank sum test to compare the 2^-ΔCt^ values between the groups.

**
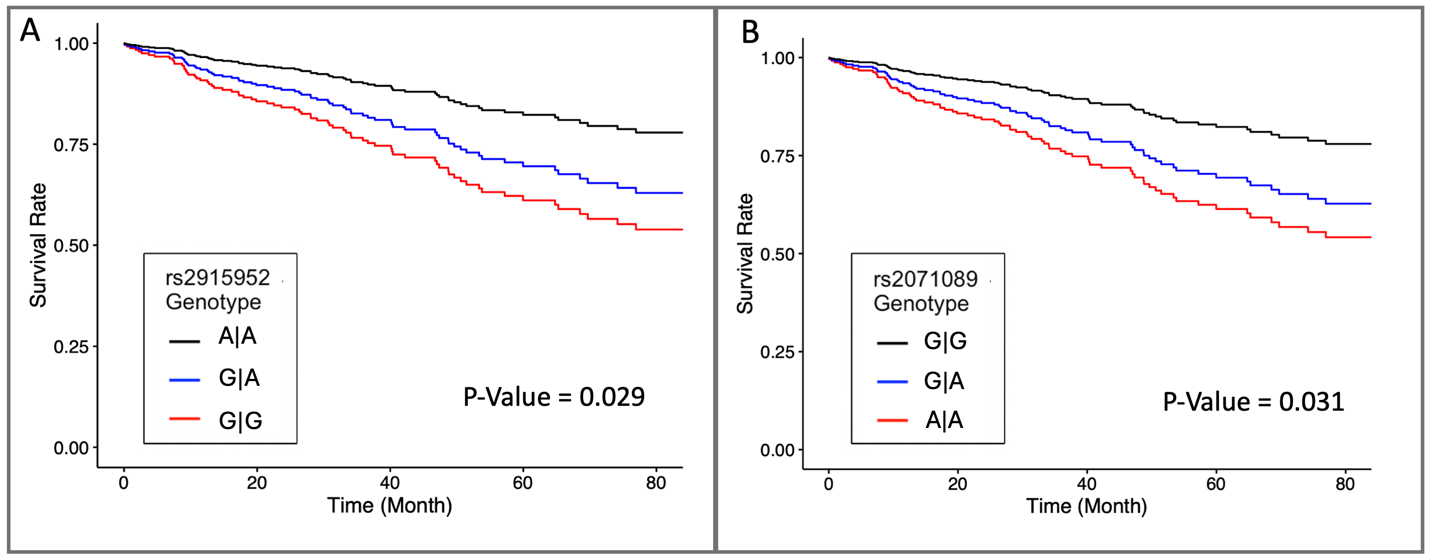
Supplementary Figure 2**: Estimated adjusted hazard curves for all-cause mortality across rs2915952 (A; N = 53 for A/A, N = 145 for G/A, and N = 129 for G/G) and rs2071089 (B; N = 52 for G/G, N = 145 for G/A, and N = 130 for A/A) genotype. Lines denote the homozygous common genotype (red), the heterozygous genotype (blue) and the homozygous variant genotype (black), respectively. *P*-values were computed using a multivariable Cox proportional hazard regression model.


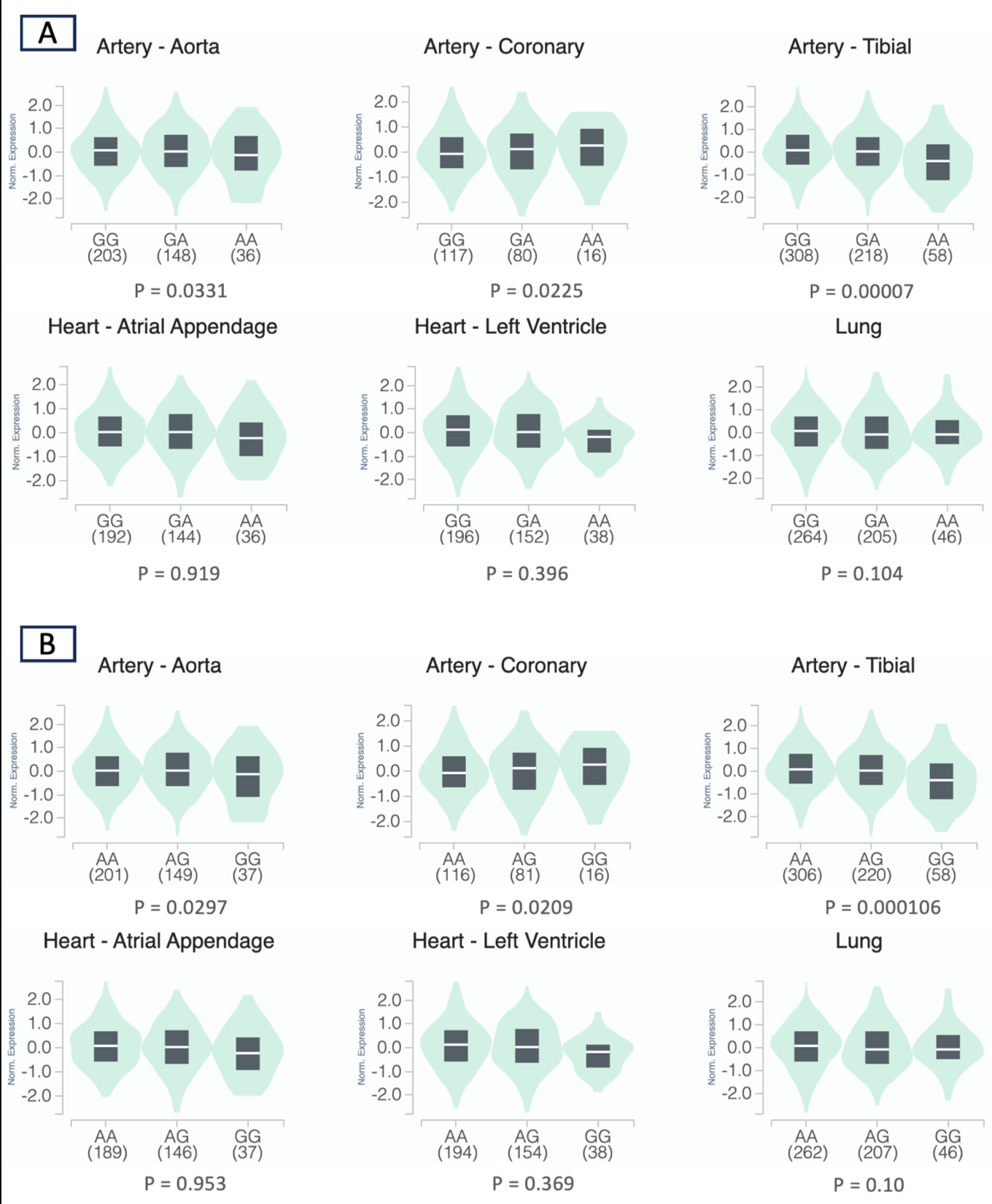


**Supplementary Figure 3:** Violin plots depicting the normalized expression levels of *RYR1* by rs2915952 (A) and rs2071089 (B) genotype using data obtained from the GTEx database (human genome build 38) ^22^. For rs2915952, the G and A alleles represent the major and minor alleles, respectively, and for rs2071089, the A and G alleles represent the major and minor alleles, respectively. The density distribution of samples within each genotype is visualized in green, while the median expression value of *RYR1* for each genotype is represented by the white line within the black box plot. Sample sizes for each genotype are included in the parentheses under the corresponding genotype.
